# Bilateral Lower Limb Amputations in a Nigerian Child Following High-Voltage Electrical Burns Injury: A Case Report

**DOI:** 10.5704/MOJ.1307.004

**Published:** 2013-07

**Authors:** EM Dim, OC Amanari, TE Nottidge, UC Inyang, A Nwashindi

**Affiliations:** Department of Orthopaedics and Traumatology, University of Uyo Teaching Hospital, Uyo, Nigeria; Department of Burns and Plastic Surgery, University of Uyo Teaching Hospital, Uyo, Nigeria; Department of Orthopaedics and Traumatology, University of Uyo Teaching Hospital, Uyo, Nigeria; Department of Orthopaedics and Traumatology, University of Uyo Teaching Hospital, Uyo, Nigeria; Department of Burns and Plastic Surgery, University of Uyo Teaching Hospital, Uyo, Nigeria

## Abstract

The human body conducts electricity very well. Direct contact with electric current can be lethal. The passage of electric current through the body is capable of producing a wide spectrum of injuries, including serious damage to the heart, brain, skin and muscles. Naked high-voltage electric cables negligently abandoned in residential, commercial and industrial areas are a recipe for disaster. This is a case report of a 5-year girl child who had bilateral lower limb gangrene following electrical burns injury. She presented with a fourday history of electrical burns injuries of both lower limbs including both gluteal regions, associated with a three-day history of fever, with full thickness burns and sepsis, ultimately leading to bilateral above knee guillotine amputations.

**Key Words:**

High-voltage electric current, bilateral lower limb gangrene, bilateral above knee amputation

## Introduction

It is common to see high-voltage electric cables haphazardly slung or running across densely populated industrial, residential, or commercial premises in our environment. Sometimes, these cables are ‘live’ with electricity even as they lie on the ground, hang negligently down from tree tops, and across farmlands, thereby posing serious safety challenges to the populace.

Electrical burns are generally uncommon in Nigeria and they constitute about 2.8% of all burns in this region. Studies in Nigeria showed that high-tension electric burns usually involve electricity workers or electrical installation vandals. High-tension electrical burns are not common among children in Nigeria. In the developed countries, electric burns are also responsible for about 3-5% of all burns unit admissions, but high-tension electrical injuries are usually from power lines and rail sources^1^,^2^,^3^,^4^.

Electrical currents can produce diverse and serious injuries to the heart, brain, skin and muscles. The extent of tissue damage following electrical injuries is related to the voltage of the current. High-voltage electrical injuries often produce severe burns and blunt trauma. Such burns are often much worse than they initially appear. Mortality depends on the electrical contact and is estimated to be 3-15%^1^,^5^.

Lack of access to prompt medical care in well equipped highly specialized units is a contributory factor to poor outcome for these patients. We present a case of bilateral lower limb gangrene in a child following high-tension electrical burn injury.

## Case Report

A five-year old girl presented at the Children emergency department of the University of Uyo Teaching Hospital, Uyo, Nigeria, in June 2012, with a four-day history of electrical burns injuries to both lower limbs and the gluteal regions, associated with a three-day history of fever. She was said to be playing outside of her mother’s shop beside a major street in Uyo metropolis, when she had stepped on a naked hightension electric cable hanging down from an electric pole. She suffered electric burn injuries to her lower limbs, buttocks, lower back, trunk, and scalp over the vertex. There was no loss of consciousness. She was taken to a nearby private hospital, from where she was referred to us on the fourth post injury day.

**Fig. 1:**
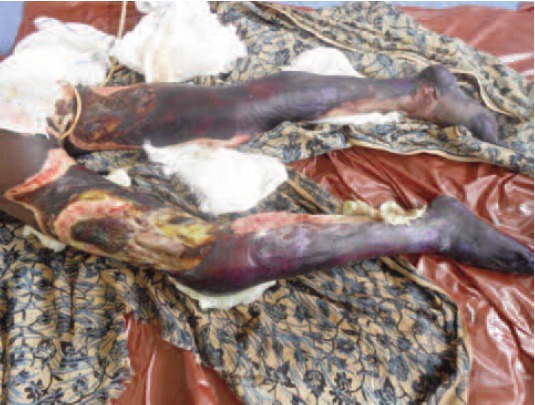
: Extensive bilateral lower limbs electrical burns injuries

On examination, she was found to be acutely ill, severely dehydrated, and with a body temperature of 38.6 degrees Celcius. She was in pain, pale, and anicteric. The heart sounds were normal and the chest was clear. Neurological assessment was normal. There were extensive full-thickness burns wounds in the lower limbs, extending posteriorly over both buttocks to the lumbosacral region. Anteriorly, the skin over the proximal half of the right thigh and proximal one third of the left thigh was spared. There was an extensive deep laceration on the lateral aspect of the distal two thirds of the left thigh, exposing a long segment of desiccated femur. There was deep wound sepsis and gangrene of the lower limbs, from the feet extending as far proximally as the junctions of the proximal and middle thighs. There was an 8cm by 6cm full thickness burn wound on the left chest wall posteriorly, and an oval burn wound on the scalp with a central scab measuring 16cm in widest diameter. The total body surface area burnt was 42% (Figure 1).

**Fig. 2:**
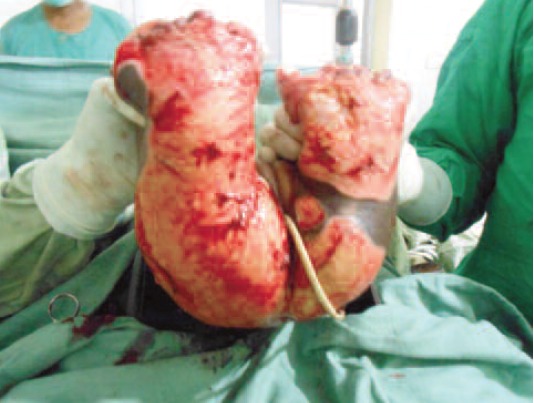
: Bilateral high above knee amputations for the electrical burns injuries.

The packed cell volume at presentation was 39%. It later dropped to 25% and then 19%. Serum electrolytes, urea and creatinine were within normal limits. The patient was appropriately resuscitated with intravenous fluid, blood transfusion and broad spectrum antibiotics. She had bilateral above knee guillotine amputation through the proximal thighs, at the level where viable tissue was encountered (Figure 2).

**Fig. 3:**
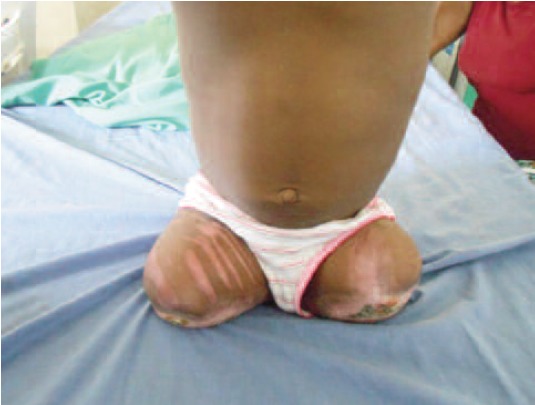
: Post operative rehabilitation of the amputee.

Postoperatively, the patient continued to receive appropriate antibiotics and blood transfusions as required. Nutritional support in the form of amino acid infusions and oral intake of high-protein and high-calorie feeds was instituted. She had daily dressings to the stump and burns wounds. The burns unit undertook subsequent wound care involving resurfacing with split skin grafting of the amputation stumps and residual burn wounds. Physiotherapy to both hips was also instituted (Figure 3).

## Discussion

Electricity, although an important commodity, has become a significant cause of injury in our society^3^. Electrical injuries are caused by the conduction of electric current through the body. The injuries could be classified into low-voltage (<1000V) and high voltage (>1000V) injuries^3^. Although both low and high-voltage electrical injuries can have devastating effects, the high-voltage electrical injuries tend to produce more extensive tissue damage. Alternating current, the more commonly distributed form, is more dangerous than direct current due to the tetanic muscle spasms it produces and this results in prolonged contact of the victim with the source. The mechanisms of injury include flash burns which results from the luminous bridging that occurs when current is shorted, and the direct conduction of the current through the patient. Indirect injuries, such as fractures either from severe muscle contractions or falls, are also common^3^. Studies in Nigeria showed that electrical injuries form a small percentage of burns admissions in this region. The high voltage electrical injuries in several instances resulted in the loss of one or more limbs. The percentage of such patients requiring amputation was as high as 86%.

Majority of high-tension electrical injuries involve electricity workers. High-tension electrical injuries resulting from contact with either fallen or low lying live high-tension cables in neighborhoods have been documented. High-tension electrical injuries make up a much smaller percentage of paediatric ICU admissions unlike adults since most of these injuries are work related. However contact with exposed high-tension cables located near residential areas as reported in this case could account for a significant proportion of these injuries in children.

In developing countries, most patients lack access to prompt medical care in well equipped highly specialized units. This was the case in our patient who presented late with thrombosis of the major vessels of both lower limbs making them non-viable. Hence she required amputation surgery for the non salvageable limbs, and the remaining wounds covered with split skin grafts. The circumstances that led to the devastating injury in this case highlight theneed to establish and enforce adequate safety regulations and enforcements. These include properly locating high-tension electrical cables to prevent accidental contact, prompt repair of fallen high-tension cables, and provision of specially designed wear and ladders for electricity workers. Appropriate education of the public would also help. These measures would reduce the incidence of high-tension electrical injuries which are mostly preventable.
